# Spatial Genome Organization and Its Emerging Role as a Potential Diagnosis Tool

**DOI:** 10.3389/fgene.2016.00134

**Published:** 2016-07-26

**Authors:** Karen J. Meaburn

**Affiliations:** Cell Biology of Genomes Group, National Cancer Institute, National Institutes of HealthBethesda, MD, USA

**Keywords:** genome organization, nuclear architecture, spatial positioning, gene positioning, disease, cancer, diagnosis

## Abstract

In eukaryotic cells the genome is highly spatially organized. Functional relevance of higher order genome organization is implied by the fact that specific genes, and even whole chromosomes, alter spatial position in concert with functional changes within the nucleus, for example with modifications to chromatin or transcription. The exact molecular pathways that regulate spatial genome organization and the full implication to the cell of such an organization remain to be determined. However, there is a growing realization that the spatial organization of the genome can be used as a marker of disease. While global genome organization patterns remain largely conserved in disease, some genes and chromosomes occupy distinct nuclear positions in diseased cells compared to their normal counterparts, with the patterns of reorganization differing between diseases. Importantly, mapping the spatial positioning patterns of specific genomic loci can distinguish cancerous tissue from benign with high accuracy. Genome positioning is an attractive novel biomarker since additional quantitative biomarkers are urgently required in many cancer types. Current diagnostic techniques are often subjective and generally lack the ability to identify aggressive cancer from indolent, which can lead to over- or under-treatment of patients. Proof-of-principle for the use of genome positioning as a diagnostic tool has been provided based on small scale retrospective studies. Future large-scale studies are required to assess the feasibility of bringing spatial genome organization-based diagnostics to the clinical setting and to determine if the positioning patterns of specific loci can be useful biomarkers for cancer prognosis. Since spatial reorganization of the genome has been identified in multiple human diseases, it is likely that spatial genome positioning patterns as a diagnostic biomarker may be applied to many diseases.

## A Shortage of Quantitative Cancer Biomarkers

Cancer is a major public health issue. In 2012 alone, across the globe approximately 14.1 million people were diagnosed with cancer and 8.2 million people lost their lives to it (Torre et al., 2015). In developed countries a third to a half of the population will be diagnosed with cancer during their lifetime (Ahmad et al., 2015; Siegel et al., 2016). Not only is cancer a leading cause of morbidity, it is also a leading cause of mortality. In fact, only heart disease accounts for more deaths than cancer; although cancer is the most frequent cause of death for 40–79 year olds (Siegel et al., 2016). Cancer is a heterogeneous collection of diseases. The most prevalent cancers are lung, breast, prostate and colorectal carcinomas, which, when combined account for ~43% of all new cancer diagnoses (Torre et al., 2015; Siegel et al., 2016). These four cancers are also leading causes of cancer-related mortality, contributing ~40% of the total (Torre et al., 2015; Siegel et al., 2016). Even cancers derived from the same organ can be quite heterogeneous from each other, and are further stratified based on morphological patterns, clinical features and/or molecular characteristics. This sub-grouping is important clinically because the appropriate treatment and the risk of recurrence or lethality differ between subgroups. For example, HER2 (also known as ERBB2) positive breast cancers have a poorer prognostic outlook than luminal breast cancers (Sorlie et al., 2001; Ferte et al., 2010), and many HER2-positive breast cancers respond well to trastuzumab (Herceptin) treatment, while endocrine therapy is an effective treatment option for the majority of luminal breast cancers (Ferte et al., 2010; Schnitt, 2010).

A key to the successful treatment of any disease is accurate diagnosis, preferably at an early stage in disease progression when treatments are generally more effective and less aggressive. However, early detection of cancer, particularly by population-based cancer screenings such as mammography or serum PSA levels, often comes at the cost of overdiagnosis (Welch and Black, 2010; Bleyer and Welch, 2012; Sandhu and Andriole, 2012). Overdiagnosis is the detection of indolent cancers that will remain asymptomatic during the patient’s lifetime and, as such, do not require treatment. Most commonly, cancer diagnosis is made following histological examination of biopsy or surgical tissue specimens. Unfortunately, in most instances there is a limit to the prognostic information that can be gleaned from histological analysis, and the pathologist is unable distinguish indolent from aggressive cancers with a high degree of certainty. Presently, due to a lack of additional markers to identify aggressive cancer, many overdiagnosed patients are being overtreated since they are receiving unnecessary therapies. Not only are these patients receiving no therapeutic benefit, as their cancer would never have significantly progressed, they are actually being harmed by the cancer treatment itself, because treatments can have both short-term and long-term effects that cause illness, reduce quality of life, create large financial burdens (for example by loss of income), and can even lead to death (Cooperberg et al., 2010; Welch and Black, 2010; Bleyer and Welch, 2012; Sandhu and Andriole, 2012). It has become a major focus of research to find markers that can distinguish between indolent and aggressive cancers, to aid the determination of the best possible treatment plan for the patient.

## Targeting the Nucleus in the Search for Clinically Relevant Biomarkers

**FIGURE 1:**
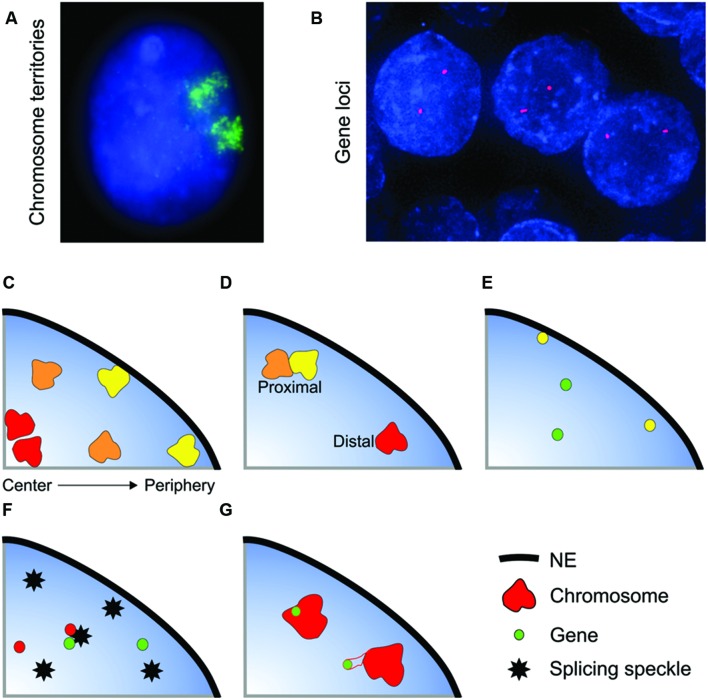
**Non-random organization of the genome. (A,B)** Whole chromosomes and genes can be visualized within interphase nuclei using FISH. **(A)** Chromosome 11 (green) in an interphase nucleus (blue) of the breast epithelial cell line MCF10A. **(B)** *SPDEF* (red) gene loci in normal prostate tissue (formalin-fixed paraffin embedded tissue) nuclei (blue). The spatial organization of the genome is characterized in several ways: **(C)** Radial positioning describes the position of a locus relative to the center and periphery of the nucleus. Gene-poor chromosomes (yellow) generally locate to the nuclear periphery, whilst gene-rich chromosomes have a preference for the nuclear interior. **(D–G)** Relative positioning describes the position of a locus relative to another nuclear landmark. Association frequencies can either be measured on a per cell basis (i.e., the number of nuclei in a population with at least one copy of a gene or chromosome in close proximity to the given landmark) or on a per allele basis. Examples of types of relative positioning include: **(D)** The position of a locus relative to another locus. The yellow and orange chromosomes are in close spatial proximity, whereas the orange and red chromosomes are distal. **(E)** Association with the nuclear envelope (NE). Some genes preferentially co-localize with the NE (yellow gene). **(F)** Association with a nuclear body such as a splicing speckle. **(G)** The position of gene relative its own chromosome territory. Some gene rich loci loop out of the bulk of the chromosome territory when highly expressed.

Molecular markers, by their very nature, tend to be highly quantifiable, and are a good potential source of diagnostic and prognostic cancer biomarkers (Ferte et al., 2010). However, few molecular markers are currently in routine clinical practice (Ferte et al., 2010). An exciting area for exploration to identify novel biomarkers is cancer-related changes in the architecture of the cell nucleus (Veltri and Christudass, 2014). This is not without precedence and changes in nuclear structure, such as nuclear size, shape, prominence of nucleoli and chromatin texture, have long been used by pathologists as part of their criteria to identify cancerous cells (Zink et al., 2004b; Veltri and Christudass, 2014). Moreover, identifying chromosomal translocations is important for diagnosing hematological cancers, as specific translocations characterize the type of leukemia or lymphoma, are associated with distinct clinical features and are often indicative of the appropriate treatment (Mitelman et al., 2007). For example, chronic myeloid leukemia is diagnosed after the cytogenetic detection of *t*(9;22)(q34;q11), the Philadelphia chromosome, which results in a *BCR*-*ABL* fusion protein. Tyrosine kinase inhibitors (Imatinib/Gleevec) inhibit the activity of the resultant oncogenic fusion protein and use of this targeted therapy results in exceptionally high rates of remission for chronic myeloid leukemia patients (Hehlmann et al., 2007). FISH, a technique used to visualize selected sequences of DNA within interphase nuclei (**Figures 1A,B**) or on mitotic chromosomes, is one method used in clinical practice to detect the presence of specific translocations (Muhlmann, 2002; Zink et al., 2004b; Hehlmann et al., 2007). FISH is also used clinically to detect other chromosomal aberrations in solid and hematological cancers, such as amplifications of the *ERBB2* locus in breast cancer, to aid diagnosis or as a prognostic marker (Muhlmann, 2002; Lambros et al., 2007; Hastings, 2010). Changes in gene expression profiles can also be useful diagnostically. For instance, there are several commercially available assays with prognostic value for various sub-types of breast cancer based on the gene expression profiles of between 2 and 97 genes (Dai et al., 2015).

Beyond gene expression changes and genomic aberrations, there are multiple other aspects of nuclear structure and function that are deregulated in cancer and could be exploited clinically. For example, alteration in nuclear shape, size, chromatin texture, global levels and patterns of heterochromatin and/or histone modifications during carcinogenesis have been shown to be predictive of cancer progression (Zink et al., 2004b; Nielsen et al., 2008; Veltri and Christudass, 2014; Hveem et al., 2016). Aberrant expression of A- and/or B- type lamins is a common feature of many types of cancer, including lung, breast, prostate, colorectal, skin, and gut carcinomas (Broers et al., 1993; Moss et al., 1999; Venables et al., 2001; Willis et al., 2008; Belt et al., 2011; Kong et al., 2012; Wazir et al., 2013; Broers and Ramaekers, 2014; Saarinen et al., 2015). The majority of lamin proteins form the nuclear lamina, which underlies the NE, and an additional pool of intranuclear lamins exists (Dittmer and Misteli, 2011). Although highly variable between individual cancers and cancer sub-types, a broad generalization suggests cancers with lower expression levels of A-type lamins tend to have poorer outcomes and more aggressive phenotypes (Belt et al., 2011; Wazir et al., 2013; Saarinen et al., 2015). This is in line with findings that cells with a deficiency in A-type lamins can migrate more easily though narrow constrictions (Davidson et al., 2014), which may aid metastatic progression, and that reduced lamin A has been linked to a higher cellular proliferation rate (Venables et al., 2001). However, the associations between reduced lamin expression and prognosis tend to be weak or based on a low number of patients, and there are conflicting studies that find higher levels of A-type lamin expression to be predictive of a poor outcome (Willis et al., 2008; Kong et al., 2012). Since difference in lamin expression can vary between sub-groups of cancers, for example between small cell lung cancer and non-small cell lung cancer (Broers et al., 1993), future studies with more defined cohorts of patients may prove enlightening. It is not only changes in expression, mis-localization of lamins to the cytoplasm (Broers et al., 1993; Moss et al., 1999), or the nucleolus (Venables et al., 2001) have also been observed in some cancer specimens. The reduction and/or loss of lamin isoforms may be an early event in carcinogenesis as, at least in cervical cancer, it can occur before neoplasia is detectable (Capo-Chichi et al., 2016). Other nuclear bodies, including the nucleolus and PML bodies, are also disrupted in cancer, with the specific aberration detected dependent on the type of cancer (Zink et al., 2004b).

While loss of genomic integrity, such as mutations, amplifications, deletions and translocations, are hallmarks of cancer and cancer progression (Mitelman et al., 2007; Hanahan and Weinberg, 2011), an additional feature of the genome, the spatial positioning of individual genetic loci within the interphase nucleus, may also have the potential to become a prominent cancer biomarker.

## Non-Random Spatial Organization of the Genome in Interphase Nuclei

The potential of the spatial organization of the genome to be used as a biomarker for cancer diagnosis has been suggested by the fact that specific genes reproducibly occupy alternative nucelar positions within cancerous tissues compared to normal, yet are positioned in very similar patterns between individuals in non-diseased cells (Meaburn et al., 2009; Leshner et al., 2016; Meaburn K. et al., 2016; Meaburn K. J. et al., 2016). Before discussing the spatial reorganization of the genome in disease states, this review will first focus on what is known about how the genome is spatially organized within interphase nuclei and its role in correct nuclear function.

The genome is highly spatially organized within the nucleus, with, for the most part, each gene and chromosome occupying preferred nuclear positions (Cremer and Cremer, 2001; Ferrai et al., 2010; Dekker and Misteli, 2015; Meaburn K. et al., 2016). Although the spatial organization of the genome is non-random it does not mean that each allele in each cell within a population has an identical positioning pattern. In fact, spatial genome organization is probabilistic in nature with considerable cell-to-cell variability, and while in the majority of cells a certain position (e.g., internal, proximal to a nuclear speckle, etc.) will be favored, within the population of cells a specific genomic region can be found in any position within the cell (Parada et al., 2003; Bolzer et al., 2005; Goetze et al., 2007; Berger et al., 2008; Tjong et al., 2012; Dekker and Misteli, 2015; Kind et al., 2015). Moreover, the two alleles within the same cell can adopt different positions (**Figures 1E–G**), for example the clustering of chromosomes 12-14-15 in mouse spleenocytes most often only involves 1 copy of each chromosome per cell, with the second copy of each of these chromosomes not clustering together (Parada et al., 2002).

The spatial positioning of genomic loci can be characterized in several ways, the most common being radial positioning and relative positioning (**Figure 1**). Radial positioning describes the position of a locus along a central axis between the nuclear periphery and the center of a nucleus (**Figure 1C**). For instance, in many cell types human chromosome 18 preferentially locates to the nuclear periphery, and, conversely, chromosome 19 is positioned centrally (Croft et al., 1999; Boyle et al., 2001; Cremer et al., 2003). On the other hand, relative positioning measures the position of a gene or chromosome relative to another nuclear landmark, such as to another gene or chromosome, association with the NE or with a nuclear body, including a transcription factory or a splicing speckle, or the position of a gene with respect to the rest of the chromosome it is located on (**Figures 1D–G**). Relative positioning can measure either the frequency of close spatial proximity (either on per cell or per allele basis) or the distance between the two objects. As an example of relative positioning, in human intermediate erythroblasts cells the α*-globin* gene locus frequently loops out of its chromosome territory (**Figure 1G**), and is often in close spatial proximity to the β*-globin* gene at splicing factor-enriched nuclear speckles (**Figure 1F**) (Brown et al., 2006). Traditionally radial and proximal positioning is measured after FISH. More recently, biochemical methods have been developed to map the proximity of a locus to either other genomic regions (a range of chromatin conformation capture techniques, e.g., 3C, 4C, 5C, GCC, HiC) or to nuclear structures (e.g., DamID, ChIA-PET) (Rodley et al., 2009; van Steensel and Dekker, 2010; Bickmore and van Steensel, 2013; Dekker and Misteli, 2015). These traditional and new methods are highly complementary, and it is their combined use that will drive forward our understanding of genome organization. Unlike FISH, the biochemical methods are population-based and the heterogeneity inherent in positioning, either between individual cells or between the two alleles of a gene within a single cell, is not captured. Conversely, as opposed to many of the biochemical methods, FISH is relatively low-throughput and candidate genes are required for analysis. Thus, there exist multiple complementary methods for examining nuclear organization. It should be noted that due to current cost and complexity, sequencing-based methodologies may less suitable at present for clinical use in countries with developing economies. DNA FISH-based microscopic methods are a potentially universal and flexible technology with relatively straightforward analysis protocols that can likely be performed in many more countries.

## Correlations Between Spatial Genome Organization and Transcription

The exact molecular mechanisms that regulate the spatial organization of the genome are currently not well elucidated. Moreover, it is not clear if the spatial organization of the genome is important to regulate proper nuclear function or if it is simply a byproduct of nuclear processes. Part of the difficulty in resolving this question is that it is virtually impossible to completely separate the various nuclear functions from each other and, therefore, determine cause and effect. It is also likely that different genes are regulated by different mechanisms; for every rule suggested to govern spatial genome organization there are multiple examples of exceptions. As such, there are multiple layers of nuclear function that ultimately may influence gene positioning in normal and diseased cells. There are several lines of evidence that suggest functional importance to spatial genome organization. The first hint comes from the fact that gene-rich regions of the genome are spatially separated from gene-poor regions (Boyle et al., 2001; Boutanaev et al., 2005; Clemson et al., 2006; Shopland et al., 2006; Simonis et al., 2006; Lieberman-Aiden et al., 2009; Boyle et al., 2011; Pichugina et al., 2016). For instance, gene-rich chromosomes are generally internally located whereas gene-poor chromosomes are enriched at the nuclear periphery (Boyle et al., 2001) and within individual chromosomes gene-poor and gene-rich chromatin are spatially separate, even at the Mb scale (Boutanaev et al., 2005; Shopland et al., 2006; Lieberman-Aiden et al., 2009). This suggests that the spatial organization of the genome is functionally important for gene expression, or is at least dictated by gene expression. This hypothesis is supported by the observation of spatial clustering of some co-regulated genes, e.g., tRNA genes (Thompson et al., 2003), KLF1-regulated genes (Schoenfelder et al., 2010), STAT-regulated genes (Hakim et al., 2013) or olfactory receptor genes (Clowney et al., 2012). Further, genomic regions that are gene-poor and/or contain mostly inactive genes tend to cluster together, for example at the nuclear lamina, around the edge of the nucleolus or with peri-centromeric heterochromatin (Pickersgill et al., 2006; Guelen et al., 2008; Nemeth et al., 2010; Peric-Hupkes et al., 2010; van Koningsbruggen et al., 2010; Kind et al., 2013; Wijchers et al., 2015). Moreover, the spatial organization of the genome is different in cells with differing gene expression profiles, for example the genome reorganizes during differentiation and is different between different cell- and tissue-types (Kosak et al., 2002; Parada et al., 2004; Solovei et al., 2009; Peric-Hupkes et al., 2010; Foster et al., 2012; Hakim et al., 2013; Rao et al., 2014; Meaburn K. J. et al., 2016). While specific loci often change nuclear positions, there is not a global spatial rearrangement of the genome between cell types. Positioning patterns are remarkably conserved between cell types and stages of differentiation, with only specific genomic regions in alternative positions. For instance, in human fibroblasts and lymphocytes most chromosomes are in similar radial positions and only the position of chromosomes 8, 20, and 21 differ (Boyle et al., 2001; Meaburn et al., 2008). Similarly, of the genomic regions that associated with the nuclear lamina in mouse embryonic stem cells and astrocytes, approximately 20% are lamina-associated in only one of the two cell types (Peric-Hupkes et al., 2010).

Most saliently, genes can relocate to different nuclear locations with changes in their transcriptional output (Fraser and Bickmore, 2007; Ferrai et al., 2010). For example, active *Gfap* and *Il4* alleles are generally more internally located than their silent counterparts (Takizawa et al., 2008a) and *CFTR*, *Mash1* and *IgH* display lower association with the nuclear periphery upon activation (Kosak et al., 2002; Zink et al., 2004a; Williams et al., 2006). Conversely, genes, such as *INO1*, *HXK1* and *GAL1* move to the NPC, at the periphery of the nucleus, upon activation (Brickner and Walter, 2004; Taddei et al., 2006; Brickner et al., 2007; Berger et al., 2008) and the “gene territories” occupied by loci such as *GAL1* increase when activated (Berger et al., 2008). It is not only changes in radial position that are detected to correlate with gene expression, for example, some highly expressed gene-rich regions of the genome, including the *major histocompatibility complex*, *epidermal differentiation complex* and *HoxB* loci loop out of the bulk of their chromosome territory when active (Volpi et al., 2000; Williams et al., 2002; Chambeyron and Bickmore, 2004) and changes to the cell-cycle phase specific clustering of genes sets in yeast correlated well with changes in gene expression, particularly for genes that down regulate during the transition of cell cycle phase (Grand et al., 2014). Not all changes in nuclear location coincide with the exact time of changes in expression. Some loci reposition before changes in gene expression, adopting poised positions in the days leading up to developmentally regulated activation of expression (Ragoczy et al., 2003; Chambeyron and Bickmore, 2004; Peric-Hupkes et al., 2010), suggesting, at least for some regions, changes in nuclear positions are not simply a consequence of the activation of gene expression. On the other hand, some genes move only after changes in expression, for example, during erythroid differentiation the mouse β*-globin* gene is activated prior to relocation away from the nuclear periphery (Ragoczy et al., 2006). In this case the movement of the locus might help modify the magnitude of transcription since expression is further increased with the repositioning (Ragoczy et al., 2006). It is not only upon activation genes alter nuclear location. Relocation to blocks of heterochromatin is associated with gene repression for some genes (Brown et al., 1997, 1999; Francastel et al., 1999; Wijchers et al., 2015); however, this is not a general rule as other silenced gene loci do not associate with blocks of heterochromatin (Moen et al., 2004; Takizawa et al., 2008a).

## Lack of Correlations Between Spatial Genome Organization and Transcription

In addition to the discrepancy amongst genes on the timing of spatial repositioning in relation to altered transcription, there are a large number of studies demonstrating that for many genes changes in nuclear position and gene expression are uncoupled (Scheuermann et al., 2004; Williams et al., 2006; Kumaran and Spector, 2008; Meaburn and Misteli, 2008; Takizawa et al., 2008b; Morey et al., 2009; Harewood et al., 2010; Hakim et al., 2011; Shachar et al., 2015). For instance, when a breast epithelial cell line, MCF10A, was grown under four different growth conditions there was no correlation between change in gene expression and change radial positioning pattern for any of the eleven genes analyzed (Meaburn and Misteli, 2008). There could be several reasons to explain this. Firstly, radial positioning is not an ideal measure to assess function (Takizawa et al., 2008b). With the exception of the NE, all radial locations within the nucleus are approximately equal with respect to function and gene expression. Transcription factories and active genes are equally distributed throughout the nucleus (Wansink et al., 1993; Takizawa et al., 2008a; Paz et al., 2015). Thus, changes in radial position do not necessary represent biologically relevant changes, as they do not correlate with moving a locus into a more or less transcriptionally active environment. Moreover, radial positioning can miss biologically relevant changes to positioning if it does not shift the locus to a different radial zone. Proximity to and association with nuclear bodies is likely to be far more important for gene expression and RNA processing than radial positioning. Indeed, for many genes changes in gene expression accompany changes in association with nuclear sub-compartments, including the lamina, nucleolus, transcription factories, heterochromatin, polycomb bodies, NPCs, Cajal bodies and nuclear speckles (Ahmed et al., 2010; Ferrai et al., 2010; Bickmore and van Steensel, 2013; Khanna et al., 2014; Dekker and Misteli, 2015; Wang et al., 2016). Furthermore, transient co-localization of X chromosomes during the establishment of X inactivation and co-localization of imprinted genes with imprinting control region loci suggest a critical role for spatial genome organization in establishing and maintaining mono-allelic expression in these regions (Fraser and Bickmore, 2007; MacDonald et al., 2016). However, radial positioning vs. relative positioning cannot be the full story in explaining the differences between the studies because some genes spatially reposition in the absence of a change in expression. For example, *Mash1* relocates to a more internal nuclear position upon activation during neuronal differentiation, however, the repositioning is not specific to *Mash1* and several chromosomally neighboring genes also reposition despite remaining transcriptionally silent (Williams et al., 2006). This is not limited to transcriptionally silent genes; active genes can also change nuclear positions without modulating transcription. As an example, all four genes (*AKT1*, *BCL2*, *ERBB2* and *VEGF)* identified to change radial position after *in vitro* induction of early breast cancer were expressed at similar levels in the normal as compared to tumor cells (Meaburn and Misteli, 2008).

Why might these differences in nuclear positioning exist between different genes? It could be that some genes are more sensitive to their nuclear position for expression than others, and the genes that require specific positioning patterns for their correct expression drive the relocation of neighboring regions by “pulling” them along. Consistent with this, when loci are forced into a different nuclear environment, either by being artificially tethered or due to a chromosomal translocation, the expression of some but not all genes is affected (Finlan et al., 2008; Kumaran and Spector, 2008; Reddy et al., 2008; Harewood et al., 2010; Wijchers et al., 2015). Additionally, the type of expression change may be an important factor. Most of the genes that have been identified to alter nuclear location in parallel with changes in gene expression are genes that are being activated or silenced during development and differentiation, i.e., are genes associated with a more or less permanent change in expression, and although these expression changes occur in a coordinated manner, it does not need to be rapid. Thus, it could be that a specific nuclear environment is required for this activation/repression, and time can be taken for a locus to be relocated to this environment, but once a gene is permanently induced or silenced the environment can change without affecting the expression of that region. In support, transient homologous pairing of *Oct4* alleles occurs during mouse ESC differentiation only as *Oct4* becomes permanently repressed (Hogan et al., 2015). It is also likely that ongoing transcription is not required to maintain spatial positioning patterns, since long-range DNA interactions of active β*-globin* and *Rad23a* genes are maintained after the inhibition of RNA polymerase II transcription (Palstra et al., 2008). Precise spatial positioning, especially radial positioning, may not have a significant role for all genes beyond switching on/off. For genes that turn on and off rapidly in response to changing environmental signals, for example upon stimulation by a hormone or stress signals, rapid induction may be enhanced by proximal spatial positioning of sets of co-regulated genes prior to the stimulation, i.e., in a permanently poised position. In keeping with this, glucocorticoid receptor responsive genes cluster in the absence of glucocorticoid, and (re)stimulation with glucocorticoid does not significantly change positioning patterns (Hakim et al., 2011). Direct evidence for the concept of a more rapid activation of expression for genes already in the correct nuclear environment comes from yeast studies. The NPC associates with active regions of the genome, with specific genes recruited to the NPC upon activation (Egecioglu and Brickner, 2011; Liang and Hetzer, 2011). Even after the stimulation has been removed and the expression from these genes has been repressed several genes, including *HXK1*, *GAL1* and *INO1*, remain at the nuclear periphery and these peripheral loci are activated more quickly upon future re-stimulation (Brickner et al., 2007; Tan-Wong et al., 2009).

## Additional Players and Functions

Additionally, remodeling of chromatin is intimately linked to gene expression. Thus, it is possible that chromatin remodelers and the resulting alterations to histones and chromatin compaction are an important part of the molecular machinery that reorganizes the genome, rather than gene transcription itself. Emerging evidence suggests this may well be the case. Decondensation of chromatin in the absence of altered gene expression, even for silent genes, is sufficient for a locus to spatially reposition (Chambeyron and Bickmore, 2004; Therizols et al., 2014). Similarly, modifications to histones are important for anchoring loci to the nuclear periphery, independently of a role in gene repression these modifications are associated with (Harr et al., 2016). For example, histone acetyltransferase SAGA (Dultz et al., 2016), histone deacetylases (Zullo et al., 2012; Demmerle et al., 2013; Dultz et al., 2016) and histone H3K9 di- and tri-methylation by histone methyltransferase (Towbin et al., 2012; Bian et al., 2013; Kind et al., 2013; Harr et al., 2015) are required to localize at least some genes to the nuclear periphery. It is most likely that chromatin remodelers work in tandem and that it is specific combinations of histone marks that are required to target a genomic region to the nuclear periphery (Harr et al., 2016). It is also likely that chromatin remodelers play a role in targeting genes within the nucleoplasm, away from the NE, since chromatin remodelers EZH2 and SUV39H1 have been demonstrated to be critical for local chromatin clustering into sub-domains (Wijchers et al., 2016) and knocking down a range of chromatin remodelers and histone modifiers affected the position of several non-NE associated genes (Shachar et al., 2015).

In addition to gene expression and chromatin state, many other nuclear factors have been linked to a role in correct spatial organization of the genome, these include actin, myosin (Chuang et al., 2006; Dundr et al., 2007; Mehta et al., 2010), NE proteins, include A-type lamins (Meaburn et al., 2007; Mewborn et al., 2010; Mehta et al., 2011; Kubben et al., 2012; Solovei et al., 2013; Harr et al., 2015), B-type lamins (Malhas et al., 2007; Ranade et al., 2016), lamin B receptor (Clowney et al., 2012; Solovei et al., 2013), emerin (Demmerle et al., 2013) and several other NE transmembrane proteins (Zuleger et al., 2013), NPC proteins (Ahmed et al., 2010; Dultz et al., 2016) and non-coding RNAs (Hacisuleyman et al., 2014).

Interestingly, different genes can be targeted to the same nuclear compartment via different protein complexes and pathways, and the use of the different mechanisms may add an additional layer of control over the speed of inducing or enhancing gene expression (Randise-Hinchliff et al., 2016). Moreover, different genes require different proteins complexes for their correct nuclear location. SiRNA screening of 669 nuclear proteins, combined with mapping the radial position of 3–4 genes by FISH, identified 50 proteins with roles in spatially organizing the genome. However, of these proteins, only knocking down one, *CACNG1*, repositioned all four genes studied. Moreover, half of the hits affected the position of only a single gene (Shachar et al., 2015).

At some level underlying DNA sequence must play a role in the spatial positioning of the genome. Transcription factors bind to specific sequences in the genome, thereby, activating a specific set of genes, and genes regulated by specific transcription factors can spatially cluster (Schoenfelder et al., 2010; Hakim et al., 2011; Hakim et al., 2013). Some specific DNA sequences have been demonstrated to have a direct role in targeting genes to specific locations within the nucleus. In *Saccharomyces cerevisiae*, two short gene recruitment sequences, within gene promotors, are required to target inducible genes to the NPC, allowing an enhanced transcriptional output (Ahmed et al., 2010). It has recently been proposed that the association between repetitive sequences, in particular homotypic associations between short interspersed retrotransposable elements (SINEs), which are enriched in gene-rich regions of the genome, or long interspersed retrotransposable elements (LINEs), which are enriched in gene-poor regions, may be a key underling factor responsible for the spatial separation of gene-rich and gene-poor regions of the genome (Cournac et al., 2016; Solovei et al., 2016). However, spatial positioning patterns cannot be an intrinsic feature of a piece of DNA, since, as discussed above, genes and chromosomes can alter position without changes in DNA sequence, e.g., between cell types or upon activation. Additionally, some human chromosomes, including chromosome X, adopt different nuclear positions when they are in a mouse nucleus compared to when they are within their endogenous human cell (Meaburn et al., 2008). It is also unlikely all repeat sequences direct spatial organization, as pericentromeric satellite repeats migrate to existing heterochromatin compartments during differentiation rather than drive the aggregation of heterochromatin (Wijchers et al., 2015).

Replication has also been linked to the spatial organization of the genome. Genomic regions alter their nuclear location as they change replication timing during development and disruptions to replication can affect the radial position of some genes (Hiratani et al., 2008; Shachar et al., 2015). Similarly, mitosis and NE reformation in early G_1_ are important for some loci to establish nuclear location, especially for gene-poor and inactive regions when associating with the NE (Bridger et al., 2000; Kumaran and Spector, 2008; Kind et al., 2013) or during muscle differentiation (Neems et al., 2016). However, once more, these cannot be general mechanisms for all genes. Firstly, several genes and chromosomes reposition as cells exit the cell cycle and become quiescent or senescent, which clearly occurs in the absence of replication, mitosis and NE reformation (Bridger et al., 2000; Solovei et al., 2004; Meaburn et al., 2007; Meaburn and Misteli, 2008). Secondly, an alternative mechanism must be required for genes that move rapidly after stimulation. For example, relocation to the NPC occurs within 15–60 min of activation in 60% of nuclei (Randise-Hinchliff et al., 2016). This timing is too fast for replication, mitosis or NE reformation to play a major role.

The spatial organization of the genome may also have a role in DNA repair, since whole chromosomes can reposition after DNA damage (Mehta et al., 2013; Ioannou et al., 2015), however, given that in mammalian cells broken DNA ends do not significantly move (Kruhlak et al., 2006; Soutoglou et al., 2007; Roukos et al., 2013), the function of this repositioning is not clear. What is clear, however, is the role the spatial organization plays in the partner choice when oncogenic chromosomal translocations form, after the DNA breaks have occurred. Translocations preferentially form between loci that are in close spatial proximity prior to damage (Lukasova et al., 1997; Neves et al., 1999; Roix et al., 2003; Lin et al., 2009; Mani et al., 2009; Mathas et al., 2009; Chiarle et al., 2011; Klein et al., 2011; Hakim et al., 2012; Roukos et al., 2013). In a similar vein, the spatial organization of the genome also helps dictate the integration sites of HIV-1 virus DNA into the human genome after infection. HIV-1 integrates preferentially into highly transcribing genes that associated with the NPC (Lelek et al., 2015; Marini et al., 2015).

Most studies on the spatial organization of the genome focus on its role for the correct function of the genome. However, there is evidence to suggest the spatial organization of the genome may have additional structural roles that aid cellular function in a non-genetic manner (Bustin and Misteli, 2016). For example, the clustering of condensed chromatin to the nuclear periphery helps the nucleus withstand mechanical forces, while the redistribution of heterochromatin to the center of the nucleus in the rod cells of nocturnal mammals aids night vision by optimizing the diffraction of light though the retina (Solovei et al., 2009; Bustin and Misteli, 2016).

While trends are emerging as to how and why the genome is spatially organized, no “one size fits all model” has yet to clearly present itself. This is not surprising given the complexity of the nucleus. Most likely it will be a combination of molecular strategies working in concert, and differing depending on the gene and the cellular context, that optimizes correct nuclear function and organization. Until recently, due to practical constraints, loci could only realistically be studied in virtual isolation. With the rapidly reducing cost of sequencing and the advent of multiple biochemical methods to analyze genome organization on a larger scale, the tools are now in place to study the spatial organization of chromatin on a genome-wide scale. It will be important to compare these positioning maps to gene expression data, epigenetic maps, proteome data, non-coding RNA, replication timings etc., in combination. Moreover, it will be important to not only ask what is changing at the level of an individual locus, it will be vital to consider the chromosomal neighborhood at large. It will be critical to study candidate regions, identified by population-based biochemical methods, in greater detail using single cell FISH analysis. This will answer questions the biochemical methods can not, such as, in how many cells is a given positioning patterns found? Do both alleles of a gene behave in the same manner? Are positioning patterns found in the population mutually exclusive at the single cell level? From here, loci or nuclear factors can then be manipulated to determine the true functional relevance, beyond correlation.

## Spatial Reorganization of the Genome in Non-Malignant Disease

**FIGURE 2:**
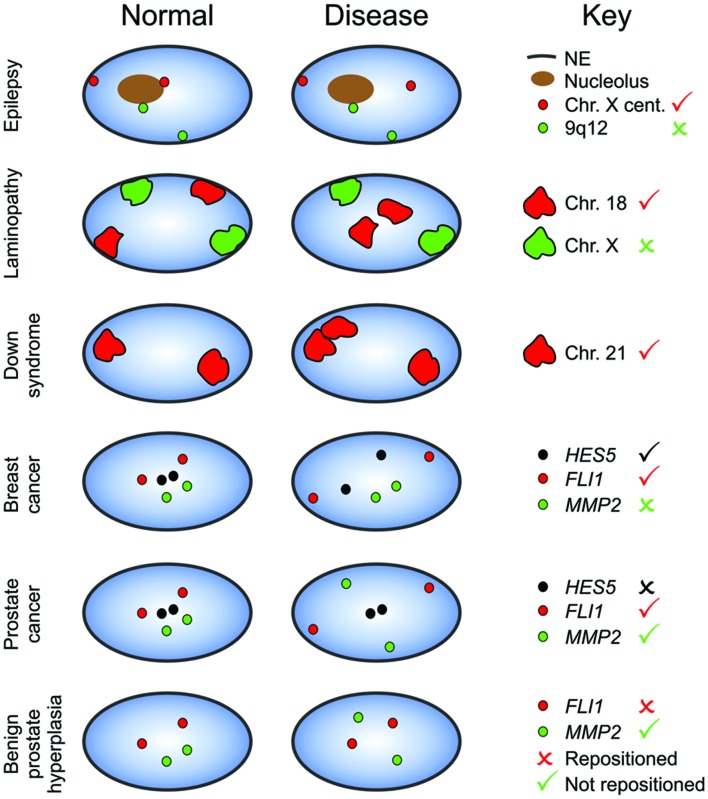
**Loci-specific reorganization of the genome in disease.** Certain loci adopt alternative nuclear positions in disease (tick) compared to normal cells, whilst the positions of other loci are conserved in disease (cross). Moreover, the repositioning of some loci is disease specific, and although a gene repositions in one disease, it may not in another disease. Blue, nucleus; NE, nuclear envelope; Cent., Centromere.

Even though many mechanistic and functional aspects of the spatial organization of the genome have yet to be resolved, these data do indicate that discrete and quantifiable changes to spatial positioning patterns exist between cells which, if they occur specifically in disease states could be exploited as biomarkers. In a wide variety of diseases, including cancer, individual genes, genomic regions and/or whole chromosomes are known to reposition (**Figure 2**) (Ferrai et al., 2010; Bridger et al., 2014; Meaburn K. et al., 2016). This phenomenon was first identified in the 1980s in brain tissues of epilepsy patients (Borden and Manuelidis, 1988). The centromere of chromosome X reproducibly redistributed away from the NE or edge of nucleoli in neurons from the epileptic seizure focus as compared to normal neurons from unaffected areas of the brain from the same individuals (**Figure 2**) (Borden and Manuelidis, 1988). This repositioning is not common to other heterochromatin regions, as neither the pericentromeric heterochromatin regions on chromosomes 1 (1q12) and 9 (9q12) nor the large heterochromatin block mapping to Yq12 reposition (**Figure 2**) (Borden and Manuelidis, 1988). Generally speaking, different loci have been positioned in different diseases, with very few loci studied in more than one affiction, making it unclear how general the disease-related repositioning of a given locus is. At least some repositioning events are different between different diseases, however. While 1q12 is not repositioned in epilepsy, in the lymphocytes from patients with endometriosis 1q12 is more peripherally located, compared to control individuals, (Mikelsaar et al., 2014) and more internally located in ICF patient lymphocytes (Jefferson et al., 2010; Dupont et al., 2012). Additional genomic regions were also found to reposition in ICF, including a reduced association of chromosome 16’s pericentromeric heterochromatin region with the NE, and an increased incidence of *human pseudoautosomal region 2* genes looping out of their chromosome territories (chromosome Y and the inactive X) (Matarazzo et al., 2007; Jefferson et al., 2010). Similarly to epilepsy, loci-specific repositioning was observed in ICF, with other regions on chromosome 1 (*BTG2*, *CNN3*, *ID3*, and *RGS1*) and the pericentromeric heterochromatin of chromosome 9 not repositioning in ICF (Jefferson et al., 2010; Dupont et al., 2012). Pericentromeric loci are not the only repeat sequences to be spatially affected in disease. Aberrant telomere clustering has been identified in several diseases, including cancer and Alzheimer’s, often due to telomere dysfunction, and the degree of clustering can reflect the aggressiveness of the disease (Chuang et al., 2004; Gadji et al., 2010; Mathur et al., 2014).

Loci-specific repositioning also occurs in laminopathies (**Figure 2**) (Meaburn et al., 2007; Taimen et al., 2009; Mewborn et al., 2010; Mattout et al., 2011; Mehta et al., 2011; McCord et al., 2013). Laminopathies are a wide ranging group of rare diseases characterized by mutations in lamin A/C, and includes Emery-Dreifuss muscular dystrophy and the premature aging disease HGPS (Dittmer and Misteli, 2011). The organization of chromosomes in proliferating laminopathy fibroblasts cells mimics that of normal quiescent cells, with chromosomes 13 and 18 shifted to a more internal position, chromosome 10 repositioning towards the nuclear periphery, however, chromosomes 4 and X remaining at the nuclear periphery (**Figure 2**) (Meaburn et al., 2007; Mewborn et al., 2010; Mehta et al., 2011). Moreover, there is evidence to suggest that the spatial reorganization of the genome in laminopathies is not limited to the repositioning of a few specific chromosomes, and may evolve as the cells increase in passage. Compared to proliferating normal fibroblasts, a dramatic genome-wide reduction in the spatial separation of active and inactive chromatin was observed in senescent HGPS fibroblasts (McCord et al., 2013). Some of these differences in interaction frequencies were senescence- rather than disease-related, since they were mostly not detected in proliferating HGPS cultures (McCord et al., 2013) yet were detected in normal senescent fibroblasts (Chandra et al., 2015). Yet, differences between normal senescent and senescent HGPS cells were detected (Chandra et al., 2015). It remains unclear how disease related these differences are, as it could be more a reflection of the type of senescence being compared. The HGPS cells used had reached (early) replicative senescence by continued culture (McCord et al., 2013), whereas senescence was induced in the normal cells by oncogene activation, which, unlike replicative senescence, forms senescence-associated heterochromatin foci (Chandra et al., 2015). The redistribution of the genome in progeria may well be progressive, as is pointed to by the findings that the abnormal clustering of centromeres associated with the *LMNA* E145K mutation only occurs in nuclei with aberrant shapes, and the prevalence increases with increased time in culture (Taimen et al., 2009).

The specific functional relevance of the spatial repositioning of the genome to the various disease phenotypes present in the different laminopathies is unclear, and appears to result more generally from the presence of aberrant A-type lamin rather than the specific disease *per se*. Generally, the spatial reorganization of the genome is very similar between different laminopathy patients, regardless of the disease or mutation present (Meaburn et al., 2007; Mewborn et al., 2010), the exception being that chromosome 13 is more peripheral in patients with Δ303 or D596N *LMNA* mutations (Mewborn et al., 2010) and the spatial organization of the genome in asymptomatic laminopathy carriers is very similar to the laminopathy patients (Meaburn et al., 2007). The release of chromosome 13 and 18 from the nuclear periphery in laminopathy fibroblasts may be linked to an altered ability of the mutant A-type lamin proteins to bind specific chromatin regions (Meaburn K. et al., 2016) and/or changes in chromatin status, since there is a reduction of heterochromatin-specific markers, including HP1α and histone H3K9 methylation, in HGPS fibroblasts (Scaffidi and Misteli, 2005), or it may be due to an uncoupling of cell-cycle regulation and spatial positioning patterns (Meaburn et al., 2007). Interesting, while few differences in positioning patterns have been detected between fibroblast cell lines of different laminopathies, the laminopathy-related spatial positioning patterns are tissue-specific, suggesting they may contribute to the disease phenotype. Chromosomal positioning abnormalities have not been detected in laminopathy patient lymphoblasts, a cell-type that does not require A-type lamins (Boyle et al., 2001; Meaburn et al., 2005). Further, in a *Caenorhabditis elegans* model of Emery-Dreifuss muscular dystrophy, a muscle-specific promotor was retained at the NE in the muscles of diseased animals, however, the release from the NE of a gut-specific promotor was not affected in gut tissue (Mattout et al., 2011).

In addition to the repositioning of gene poor regions of the genome in disease, several genes have been identified to occupy disease-specific positions. In benign prostate hyperplasia tissues *MMP2* shifts to a more peripheral position than normal prostate tissue (**Figure 2**) (Leshner et al., 2016). Benign prostate hyperplasia is not associated with a general reorganization of the genome, since the positions of *MMP9* and *FLI1* are unaffected (**Figure 2**) (Leshner et al., 2016). In lymphocytes from Down syndrome patients, two (or three) copies of chromosome 21 clusters together more frequently than in diploid control cells (**Figure 2**) (Paz et al., 2013, 2015). Moreover, *SOD1*, which is located on chromosome 21, is more peripherally located in Down syndrome (Paz et al., 2015). This altered position is not simply a consequence of the presence of an additional chromosome 21, however, as the radial position of *DYRK1A*, which also maps to chromosome 21, is unaffected (Paz et al., 2015). It is not only chronic diseases that are associated with a spatial reorganization of the genome. Short-term repositioning of some, but not all, genes or chromosomes occurs after viral or parasitic infection (Li et al., 2010; Knight et al., 2011; Arican-Goktas et al., 2014). Chromosome 17, but not 18, relocates to a more peripheral position in human lymphocytes within 24 h of Epstein-Barr virus infection (Li et al., 2010). Similarly, dynamic radial repositioning of *actin*, *ferritin* and *hsp70*, occurs in both cultured snail cells and in intact live snails within hours of active parasitic infection, with the repositioning correlating to changes in gene expression (Knight et al., 2011; Arican-Goktas et al., 2014).

In most cases the link between gene repositioning in disease and the disease phenotype has not clear. In a few cases a direct link between altered positioning patterns and disease relevant gene expression has been demonstrated. The spatial clustering of *FTO* and *IRX3* is implicated in obesity, by altering the expression of *IRX3* expression and consequently mis-regulating body mass (Smemo et al., 2014). Local disruptions to hierarchical genome organization can also be important for disease progression. High-resolution mapping of the 3D genome has revealed that beyond a spatial separation of gene-rich and gene-poor chromatin, chromosomes further fold into a succession of ten to hundreds of kb long self-associating globular domains, known as TADs (Dixon et al., 2012; Nora et al., 2012; Sexton et al., 2012). Individual TADs are stably conserved between species and cell types and are separated by sharp boundaries that are enriched in histone marks associated with active genes, SINEs, binding sites for the chromatin-binding protein CTCF and housekeeping genes (Dixon et al., 2012). TAD boundaries are believed to be important for gene regulation, by acting as insulators, inhibiting the expansion of either heterochromatin or active chromatin into a neighboring TADs and by increasing the frequency of specific regulatory interactions by confining certain promoters and enhances into a single TAD (Dixon et al., 2012; Sexton et al., 2012; Narendra et al., 2015). Consentient with this, genes within TAD domains are co-expressed during differentiation and disruption of TAD boundaries results in a partial or complete fusion of neighboring TADs leading to altered gene expression and in some cases, disease phenotypes (Nora et al., 2012; Lupianez et al., 2015; Narendra et al., 2015; Hnisz et al., 2016). Disruption to the TAD boundary near the *EPHA4* locus by deletion, duplication or inversion of DNA sequences at the boundary results in limb deformation syndromes (Lupianez et al., 2015). In normal limb development *EPHA4* is active but genes in neighboring TADs are silent. The loss of the TAD boundary allows enhancer-promoter interactions between regulatory sequences normally separated into different TADs, and consequently results in the spuriously activation of the neighboring genes in the mis-fused TAD (Lupianez et al., 2015). Similarly, cancer-associated micro-deletions or mutations at TAD boundaries can activate oncogenes, and may represent a common mechanism of gene mis-regulation in multiple cancers (Hnisz et al., 2016). The study of TADs is still in its infancy, and most likely disruption to TAD boundaries will be detected as functionally important in many other diseases.

## Spatial Genome Organization in Cancer

Given the urgent need for additional diagnostic and prognostic tests for cancer, reproducible differences in the spatial positioning patterns of the genome detected between normal/benign tissues and cancer, or between different cancer subtypes could be invaluable clinically. As with non-malignant disease, some genes and chromosomes occupy distinct nuclear positions in cancerous cells compared to their normal or benign counterparts (**Figure 2**) (Cremer et al., 2003; Murata et al., 2007; Meaburn and Misteli, 2008; Meaburn et al., 2009; Wiech et al., 2009; Zeitz et al., 2013; Leshner et al., 2016; Meaburn K. J. et al., 2016). For example, in breast cancer cell lines there is a significant change to the set of genes that are close spatial neighbors of *IGFBP3* (Zeitz et al., 2013). Moreover, ten genes (*FLI1, HES5, MMP9, HSP90AA1, TGFB3, MYC, ERBB2, FOSL2, CSF1R* and *AKT1*) have been identified to reproducibly radially reposition in epithelial cells of breast cancer tissues (Meaburn et al., 2009, Meaburn K. J. et al., 2016) and *FLI1, MMP9* and *MMP2* occupy alternative radial locations in prostate cancer (Leshner et al., 2016). Also in line with non-malignant diseases, and despite the high level of genomic instability associated with many cancers, which could conceivably be expected to result in a large-scale disruption to the spatial organization of the genome, the repositioning is loci-specific, with a general conservation of spatial positioning patterns in cancer, with the majority of genes and chromosomes not altering nuclear position (Kozubek et al., 2002; Parada et al., 2002; Cremer et al., 2003; Meaburn and Misteli, 2008; Meaburn et al., 2009; Timme et al., 2011; Zeitz et al., 2013; Leshner et al., 2016; Meaburn K. J. et al., 2016). For example, 44 of 47 (94%) genes spatially mapped in human prostate tissues do not alter radial position in prostate cancer and the radial positions of 13 out of 23 (57%) genes are conserved in breast cancer (Meaburn et al., 2009; Leshner et al., 2016; Meaburn K. J. et al., 2016).

Unlike laminopathies, the patterns of spatial reorganization differ between different cancers. While the radial positions of chromosomes 18 and 19 are affected in many cancer types, including thyroid, colon and cervical cancer, with a tendency for chromosome 18 to become more internally located and chromosome 19 to shift away from the center of the nucleus (Cremer et al., 2003; Murata et al., 2007; Wiech et al., 2009), in some cervical cancers, chromosome 18 becomes more internally located (Wiech et al., 2009). Moreover, of the eleven genes so far reported with altered radial position in either breast or prostate cancer, only *MMP9* and *FLI1* robustly reposition in both types of cancer (**Figure 2**) (Leshner et al., 2016; Meaburn K. J. et al., 2016). This suggests cancer-type specific repositioning, with different sets of genes repositioning in breast and prostate cancer. Similarly, *BCL2* repositions in a BCL2-positive cervical squamous carcinoma, but not in a BCL2-negative cervical squamous carcinoma (Wiech et al., 2009), nor in breast (Meaburn et al., 2009) or prostate cancer (Leshner et al., 2016). When taken together with the findings discussed above that a genomic region can behave differently, with respect to repositioning between different non-cancerous diseases, it suggests that the overall organization of the genome in disease is, mostly, specific to the disease and not a consequence of a more general “stress” response. In keeping with this, of the genes that occupy distinct radial positions in breast and/or prostate cancer, only *MMP2* and *ERBB2* are also commonly repositioned in non-malignant breast or prostate disease; but even for these gene it cannot be a general disease response as *MMP2* repositions in prostate but not breast cancer and visa versa *ERBB2* does not reposition in prostate cancer (**Figure 2**) (Meaburn et al., 2009; Leshner et al., 2016; Meaburn K. J. et al., 2016). Thus, there appear to be an exploitable minority of gene repositioning events that could be used to distinguish cancerous tumors from normal tissue and between different cancer types.

It is interesting to note that many of the factors implicated in influencing gene positioning patterns, as discussed earlier in this review, are often altered in cancer, e.g., lamins, gene expression, proliferation status, chromatin status and histone modifications. However, the mechanisms involved in the mis-organization of the genome in cancer are unclear. It would seem that, for the radially repositioned genes at least, transcription of the locus itself is not the key to the repositioning. No correlation was found for the repositioning of a given gene and its transcriptional output in a model of early cancer (Meaburn and Misteli, 2008). Moreover, cancer-associated genes, that are known to have altered expression in cancer, often did not reposition in breast or prostate cancer (Meaburn and Misteli, 2008; Meaburn et al., 2009; Leshner et al., 2016). Even for the cancer-associated genes that do reposition in cancer, it may not be altered transcription of the loci that is driving the alternative positioning pattern. For example, although elevated expression of *MMP9* and *MMP2* is a marker of poor prognosis in prostate cancer, and linked to metastasis, their expression is from tumor-associated stroma cells and not from the cancerous epithelial cells, where the gene repositioning was detected (Egeblad and Werb, 2002; Leshner et al., 2016). This does not rule out gene expression as a factor in repositioning, and further studies are required that measure gene expression changes in extended chromosomal regions of the repositioned gene. However, it does suggest that clinical diagnostic tests based on spatial gene positioning would be independent of gene expression and would not be a more convoluted way to get to the same result as a gene-expression based diagnostic test, and instead would be an independent biomarker that could be used in combination with existing diagnostic and prognostic strategies.

An increase in proliferation is a hallmark of cancer yet spatial positioning patterns are can also be influenced by proliferation. Again, the spatial reorganization of the genome in cancer seems to be independent of this, and does not seem to simply be a reflection of a transition from quiescent normal breast or prostate tissue to a proliferating tumor. The spatial genome organization patterns in cancer cells do not mimic proliferating (or quiescent) breast epithelial cells (Meaburn and Misteli, 2008). This is not surprising, given the fact that the majority of cells within a cancerous tumor are actually not proliferating. Ki-67 is commonly used as a marker of cellular proliferation, since it is present in all stages of the cell cycle with the exception of G_0_. Breast cancers that contain at least 30% Ki-67 positive cells are classified as highly proliferative cancers, and the majority of breast cancers, almost 60%, have only 10% or less of cells positive for Ki-67 (de Azambuja et al., 2007; Jonat and Arnold, 2011). In fact, no feature of a genomic locus has yet been identified that enables the prediction of whether a gene will reposition or not in cancer (Meaburn et al., 2009; Leshner et al., 2016; Meaburn K. J. et al., 2016), again suggesting spatial positioning patterns will be independent of other biomarkers.

## Clinical Biomarker Potential

Regardless of why the genome is spatially reorganized in the nuclei of cancer cells, the small scale studies so far performed suggest that these differences in positioning patterns have the potential to be exploited for diagnostic use (Meaburn et al., 2009; Leshner et al., 2016; Meaburn K. J. et al., 2016). Distinguishing cancer from normal tissue by spatial gene positioning patterns could practically be integrated into clinical use because it requires only a small tissue sample for analysis (100–1000s of cells), meaning it will not require additional invasive procedures for the patient. Moreover, FISH is currently already used in diagnostic labs, thus, the infrastructure for such analysis already exists in the clinic, at least in many developed countries. It remains to be seen the practicality of such a diagnostic technology for all developing economies. Importantly for clinical applications, for most genes tested, at least in the small scale studies so far performed, false positive rates were low since for the vast majority of genes spatial positioning patterns are highly similar between individuals in non-diseased tissue (Borden and Manuelidis, 1988; Wiech et al., 2005; Murata et al., 2007; Meaburn et al., 2009; Leshner et al., 2016; Meaburn K. J. et al., 2016) and repositioning events did not generally occur in non-malignant breast and prostate disease (Meaburn et al., 2009; Leshner et al., 2016; Meaburn K. J. et al., 2016). There were a few notable exceptions, *MMP2* and *ERBB2* repositions in both cancer and non-malignant disease (**Figure 2**), and the radial position of a few genes, such as *CCND1* and *TIMP3*, were highly variable between normal prostate tissues. This highlights that each candidate marker gene must be rigorously tested to eliminate genes with high false positive rates, as is standard practice for any marker brought to the clinic. Critically, particularly when genes are used in combination, spatial positioning patterns can detect breast or prostate cancer with high accuracy (Meaburn et al., 2009; Leshner et al., 2016; Meaburn K. J. et al., 2016). When genes are used as markers singularly, between 64 and 100% of samples are correctly identified as cancer based on radial positioning patterns. Multiplexing genes improved the accuracy of cancer detection for genes with lower sensitivities, reducing false negative rates (Meaburn et al., 2009; Leshner et al., 2016; Meaburn K. J. et al., 2016). Repositioning events are independent of variations in copy number and even in the background of highly variable, and in some cases large scale, genomic instability between cancer specimens, it is possible to use gene positioning to accurately distinguish cancers from control tissue-specimens (Meaburn and Misteli, 2008; Meaburn et al., 2009; Leshner et al., 2016; Meaburn K. J. et al., 2016).

Although these studies represent the largest analysis of genome organization in cancerous tissues performed to date, the eleven genes identified as potential breast or prostate cancer biomarkers were analyzed in a low number of individuals (11–19 cancers, 6–10 normal tissues and 5 non-malignant disease tissues), in tissues that were known to be cancerous or benign and were performed by the same group (Meaburn et al., 2009; Leshner et al., 2016; Meaburn K. J. et al., 2016). Moreover, these studies were not performed on tissue biopsies. This makes comparing the accuracies of gene-positioning based diagnostics with current diagnostic strategies impossible currently. The accuracy of current diagnostic tests reported are based on 100s and 1000s of biopsy specimens, from multiple testing sites, and will include sampling errors (e.g., no cells in the sample, the biopsy missing the tumor and collecting only normal tissue, the protocol failing to stain etc.). Larger scale follow-up studies are required to assess the sensitivity and specificity of these spatial positioning gene markers, to determine if they are strong enough for clinical use. It will be important to compare gene-positioning biomarkers to existing diagnostic methodologies and to establish if using the various methods in combination, or alone, improves diagnostic accuracy.

## Prognostic Potential for Spatial Genome Organization?

Additionally, the original studies were not designed to determine if the spatial organization of the genome in cancer has prognostic value. Yet prognostic markers are the most urgent cancer biomarkers required to distinguish aggressive cancer from indolent, thereby reducing overtreatment caused by over-diagnosis. It is difficult to accurately assess the current level of overdiagnosis, since directly measuring overdiagnosis involves monitoring the progress of the cancer and the cause of death of cancer patients, without treating the individuals and is also dependent on the precise definition of “clinically relevant” cancer. Nevertheless, for breast and prostate cancer, estimates suggest overdiagnosis is in the range of 22–67% of cancers (Draisma et al., 2009; Welch and Black, 2010; Bleyer and Welch, 2012; Sandhu and Andriole, 2012; Miller et al., 2014). Using prostate cancer mortality as an endpoint, as many as 84% of PSA screen-detected prostate cancers may be overdiagnosed (McGregor et al., 1998). Breast and prostate cancers are highly prevalent, with almost 1.7 million women and 1.1 million men worldwide diagnosed with these cancers, respectively, in 2012 (Torre et al., 2015). In the USA, where population-based screening for breast and prostate cancer is common, over 240,000 women and more than 180,000 men per year are diagnosed, respectively (Siegel et al., 2016). Given these numbers, even taking the low estimate of overdiagnosis of 1 in 5 cancers additional prognostic markers could benefit 100s of 1000s of patients every year and reduce health care costs considerably.

Histological assessment of tissue specimens can be beneficial in the clinical setting to reduce overtreatment. For example, pathologists classify histological patterns within prostate cancer tissue according to the Gleason grading system, which broadly correlates with the differentiation status of the cancerous tissue (Montironi et al., 2005; Epstein et al., 2016). Gleason scores, particularly when used in combination with other clinical factors, including PSA level and clinical tumor stage, are the strongest markers of prostate cancer prognosis and progression currently available (D’Amico et al., 1998; Montironi et al., 2005; Cooperberg et al., 2009; Chang et al., 2014; Epstein et al., 2016). Part of the strength of the Gleason scoring system is that it takes into account intra-individual tumor heterogeneity (Van der Kwast, 2014; Epstein et al., 2016). A low Gleason score (Gleason score 6 or below) is highly indicative of an indolent cancer, and often monitoring the tumor (watchful-waiting/active surveillance) is recommend in place of treatment (Thompson et al., 2007). However, additional markers are required as both under- and over-treatment remain an issue (Cooperberg et al., 2010). Additionally, histological analysis of tissues is, by its very nature, subjective, which can lead to inter- and intra-observer variations. Taking the example of prostate cancer again, two pathologists will agree on the Gleason score of an individual cancer tissue 36–81% of the time (Montironi et al., 2005). Similarly, the same pathologist will derive an identical Gleason score for the same tissue specimen 43–78% of the time (Montironi et al., 2005). Most frequently, the difference in score varies by a single Gleason grade, suggesting these small variations would make little difference to most patients. However, for men with Gleason score 6 or 7 cancers this could mean the difference between receiving treatment or not. The addition of quantitative diagnostic/prognoistic makers, to be used alongside histological analysis, would reduce inter- and intra-observer variations, and thus reduce overtreatment.

Given the fact that the position of a gene can vary between cancers derived from the same organ, and between cancers from different organs (Meaburn et al., 2009; Wiech et al., 2009; Leshner et al., 2016; Meaburn K. J. et al., 2016), it suggests the possibility that the spatial organization of the genome can be used to sub-type cancers for prognostic purposes. It is “simply” a matter of finding the right genes. Due to the selection criteria to identify a diagnostic spatial positioning biomarker, which is required to repositioning in the majority of prostate cancer specimens (compared to normal), it is likely different gene loci will be useful prognostics biomarkers than for diagnostic markers. For example, *FLI1* repositions in 100% (10/10) of breast cancers and 92.9% (13/14) of prostate cancers (Leshner et al., 2016; Meaburn K. J. et al., 2016), rates far too high to suggest it can be used to stratify indolent and aggressive cancers. Future screening studies are required, using a highly defined set of indolent and aggressive cancers. Since spatial reorganization of the genome has been identified in multiple human diseases, it is likely that the same principle of using spatial genome positioning patterns as a diagnostic biomarker could be applied to any disease.

## Summary

The genome is highly spatially organized, and reorganizes in disease. Accumulating evidence suggests that these repositioning patterns can be exploited for diagnostic use. Currently, there is no way to predict which genes will reposition in cancer. As we gain a better understanding of how spatial positioning is regulated in a normal cell, it may become easier to predict regions that will be diagnostically interesting. The reverse is also true. Comparing positioning patterns between normal and diseased cells will inform our understanding of spatial genome organization. While it is exciting that proof-of-principle studies have identified spatial positioning of the genome as a potentially sensitive and specific biomarker of cancer, much work is yet to be done. Large-scale follow-up studies are required to determine the feasibility and usefulness of spatial genome organization-based diagnostic in the clinical setting. Moreover, while additional diagnostic tests have their use, there is urgent need for biomarkers that can distinguish aggressive cancers from indolent ones. These markers would have a tremendous impact on cancer patients, by guiding clinicians to the best treatment regime for an individual and reducing both under and over-treatment. As such, as future studies relating to genome organization for clinical use should focus on prognostics rather than simply diagnostics.

## Author Contributions

KM wrote the review and prepared the figures.

## Conflict of Interest Statement

The author of this article has US Patent App. 13 062,247, European patent number 2321428. Method for detection of cancer based on spatial genome organization. Tom Misteli and Karen J. Meaburn (2009). The author declares that the research was conducted in the absence of any commercial or financial relationships that could be construed as a potential conflict of interest.

## Abbreviations

FISH: fluorescence *in situ* hybridization
HGPS: Hutchinson-Gilford progeria syndrome
ICF: immunodeficiency, centromere instability and facial anomalies syndrome
NE: nuclear envelope
NPC: nuclear pore complex
PSA: prostate-specific antigen
TAD: topologically associating domain
